# Effect of Chronic Administration of Low Dose Rapamycin on Development and Immunity in Young Rats

**DOI:** 10.1371/journal.pone.0135256

**Published:** 2015-08-06

**Authors:** Zhenya Lu, Furong Liu, Linglin Chen, Huadan Zhang, Yuemin Ding, Jianxiang Liu, Michael Wong, Ling-Hui Zeng

**Affiliations:** 1 Department of Internal Medicine, the First Affiliated Hospital, School of Medicine, Zhejiang University, Hangzhou, Zhejiang, China; 2 Department of Pharmacy, School of Medicine, Zhejiang University City College, Hangzhou, Zhejiang, China; 3 Department of Neurology and the Hope Center for Neurological Disorders, Washington University School of Medicine, St. Louis, Missouri, United States of America; Van Andel Institute, UNITED STATES

## Abstract

Mammalian target of rapamycin (mTOR) regulates cell growth, cell differentiation and protein synthesis. Rapamycin, an inhibitor of mTOR, has been widely used as an immunosuppressant and anti-cancer drug. Recently, mTOR inhibitors have also been reported to be a potential anti-epileptic drug, which may be effective when used in young patients with genetic epilepsy. Thus, a suitable dose of rapamycin which can maintain the normal function of mTOR and has fewer side effects ideally should be identified. In the present study, we first detected changes in marker proteins of mTOR signaling pathway during development. Then we determined the dose of rapamycin by treating rats of 2 weeks of age with different doses of rapamycin for 3 days and detected its effect on mTOR pathway. Young rats were then treated with a suitable dose of rapamycin for 4 weeks and the effect of rapamycin on mTOR, development and immunity were investigated. We found that the expression of the marker proteins of mTOR pathway was changed during development in brain hippocampus and neocortex. After 3 days of treanent, 0.03 mg/kg rapamycin had no effect on phospho-S6, whereas 0.1, 0.3, 1.0 and 3.0 mg/kg rapamycin inhibited phospho-S6 in a dose-dependent manner. However, only 1.0 mg/kg and 3.0 mg/kg rapamycin inhibited phospho-S6 after 4 weeks treatment of rapamycin. Parallel to this result, rats treated with 0.1 and 0.3 mg/kg rapamycin had no obvious adverse effects, whereas rats treated with 1.0 and 3.0 mg/kg rapamycin showed significant decreases in body, spleen and thymus weight. Additionally, rats treated with 1.0 and 3.0 mg/kg rapamycin exhibited cognitive impairment and anxiety as evident by maze and open field experiments. Furthermore, the content of IL-1β, IL-2, IFN-γ, TNF-α in serum and cerebral cortex were significantly decreased in 1.0 and 3.0 mg/kg rapamycin-treated rats. The expression of DCX was also significantly decreased in 1.0 and 3.0 mg/kg rapamycin-treated rats. However, rats treated with 1.0 mg/ kg rapamycin exhibited fewer and milder side effects than those treated with 3.0 mg/kg. In summary, all these data suggest that there is not a rapamycin dose that can inhibit mTOR for epilepsy without causing any side effects, but 1 mg /kg may be the optimal dose for young rats for suppressing mTOR with relatively few side effects.

## Introduction

Epilepsy is the third most common major neurological disease characterized by recurrent, unprovoked seizures. It affects about 50 million people around the world and is increasingly recognized as a disease that results in a range of comorbidities [1,2]. However, currently available drugs suppress seizures but do not cure epilepsy, so that many patients require life-long treatment with medication, and suffer from the side effects of the drugs, such as cognitive impairment, psychiatric problems, hepatic dysfunction and hematopoietic disorders [3,4].

In attempting to develop more effective drugs for epilepsy, the mammalian target of rapamycin (mTOR) signaling pathway has recently been investigated as a regulator of epileptogenesis [5–8]. mTOR pathway receives information from nutrients, growth factors, cytokines, and hormones through tyrosine kinase receptors, and plays an essential role in cell growth, differentiation, proliferation, and protein synthesis via phosphorylation of a number of translational regulators such as ribosomal S6 kinase. Among them, mTOR, a common protein kinase, is the key target protein kinase implicated in a large variety of physiological functions [9,10]. The relationship between mTOR pathway activation and epilepsy has been first implicated in genetic epilepsy using transgenic knockout mouse models of tuberous sclerosis complex and PTEN [5,6], and has also been examined in acquired epilepsy in animal models of temporal lobe epilepsy induced by kainic acid (KA) or pilocarpine [7,8]. Hyperactivation of mTOR pathway has also been established in hypoxia-induced neonatal seizures in animal models [11,12].

Rapamycin, a FDA-approved mTOR inhibitor, has been thoroughly studied in models of epilepsy. Treatment with rapamycin, given either to fetal or neonatal knockout mice, or either as a pretreatment or post treatment after status epilepticus, has reduced seizure frequency or prevented spontaneous seizures [5–8]. However, the effect of rapamycin in preventing epilepsy appears to be dependent on its long-term administration starting at a very early age in genetic epilepsy. Thus, its potential for chronic side effects is a major concern. Here in the present study, we first assessed the changes in critical downstream and upstream target proteins of mTOR signaling in the developing brain in normal rats. Next, we determined the effect of different doses of rapamycin on the phosphorylation of key protein ribosomal S6. Finally, we evaluated its effect on growth, development, immunity and cognition after a relatively long term administration of rapamycin in young rats.

## Materials and Methods

### Animals and drug treatment

Sprague-Dawley rats of 2 weeks of age were purchased from Shanghai Slac Laboratory Animal Corporation (certificate: SCXK 2007–0005). Both male and female rats were used. The rats were housed at Zhejiang University Animal Care Facility according to the institutional guidelines for laboratory animals. All animal experiments were performed in accordance with guidelines approved by Zhejiang University Institutional Animal Care and Use Committee. The protocol was approved by the Committee on the Ethics of Animal Experiments of the Zhejiang University (Permit Number: ZJU2015-489-02). All efforts were made to minimize pain and suffering.

Rapamycin obtained from LC Laboratories (Woburn, MA, U.S.A.) was prepared and stocked as previously reported [5]. It was initially dissolved in 100% ethanol, stored at 20°C, and diluted immediately before use in a vehicle solution containing 5% Tween 80, 5% PEG 400, and 4% ethanol (Sigma, St. Louis, MO, U.S.A.). For the short-term treatment, rats were injected i.p. once a day for 3 days at different doses. For long-term treatment, rats were injected once a day for 4 weeks. Control rats received corresponding injections of vehicle in all experiments. We tested separate “short-term” and “long-term” treatment paradigms, based primarily on differences in mTOR inhibition.

### Western blot analysis

Rats were sacrificed at pre-determined time points for Western blot analysis of mTOR pathway activation. Standard methods were used as described previously [7]. In brief, total proteins extracted from temporal neocortex and hippocampus were separated by sodium dodecyl sulfate polyacrylamide electrophoresis and transferred to nitrocellulose membrane. Neocortex and hippocampus were assayed, in correlation with the behavioral studies on cognitive function. After blocking with 5% skim milk, the membranes were incubated with the primary antibodies: phospho-Akt (Ser473,) phospho-mTOR (Ser2478) and phospho-S6 (Ser240/244) (1:1,000; Cell Signaling Technology, Beverly, MA, U.S.A.) followed by peroxidase conjugated anti-rabbit secondary antibody (1:5000, pierce, Rockford, IL). Protein bands were visualized with enhanced chemiluminescence reagent (Pierce). The membranes were reprobed and incubated with the rabbit Akt, mTOR and S6 antibody (1:1,000; Cell Signaling Technology). After measurement of the intensity of each lane in each blot by ImageJ (NIH, Bethesda, MD, U.S.A.), the ratio of p-S6 to total S6 was calculated and normalized to the control group. Each experiment was conducted at least three times.

### Morris water maze test

All behavioral experiments were conducted the next day after 4 weeks of daily rapamycin treatment. Morris water maze was conducted as described previously [13]. Briefly, a platform of 10 cm in diameter was submerged 1 cm beneath the surface and located at a fixed position and remained constant throughout the training period. Swimming activity of each rat was monitored using a video camera mounted over head (Sony, Tokyo, Japan) and automatically recorded via a video tracking system. Rats were trained for 4 days before testing. During the four consecutive training days, rats were first placed in the platform for 10 s, and then randomly placed in 4 different quadrants. Recording was stopped 10 s after the rats reached the platform. Rats were directed to the platform if they did not find it within 60 s and stayed there for 10 s. Each rat was given 4 trials per day and each trial was separated by 1 h. On the 5th day, the rat was placed in the quadrant diagonally opposite from the previous platform location and the time of reaching the platform was recorded as escape latency. Swimming distance and swimming speed were also analyzed.

### Y- maze

The procedure was described in detail previously [14]. The Y-maze used in the present study was 50 cm long and 16 cm wide with walls 14 cm high.The floor and walls were constructed from dark opaque polyvinyl plastic. A signal lamp was placed at the end of each arm and a copper shock grid on the bottoms of the maze. The arm with the light on (bright arm) indicates the safe area without footshock. The safe arm and the unsafe arm were set randomly. The correct response was considered when the rats directly ran to the bright arm without hesitating after changing the safe and unsafe arms in the Y-maze. Rats were pretested 20 times with the Y-maze for 4 days. On the 5^th^ day, rats were tested for 20 times and the correct times, the times of active escape and the time to the safe area were recorded. All tests were carried out in a dark and quiet small room.

### Open-Field test

Locomotor activity and exploratory behavior were evaluated using the open field test. It was performed in an experimental chamber, which consisted of a 60 x 60 x 20 cm^2^ of wooden box. All four chamber walls and the floor of the box were painted black. Rats were placed into the box and allowed to explore the apparatus for 5min. Movements were recorded by camera and data were analyzed using Noldus software. The number of rearings, the number of crossings (number of squares crossed), the duration of immobility periods (in s), the retention time in middle field (in s), and number of fecal pellets were analyzed [15]. When changed to another rat, the chamber was cleaned with Clidox (PRL Pharmacal Research Laboratories, Inc., Naugatuck, CT, 1:18:1) followed by 70% ethanol to assure the chamber was dry and to eliminate animal odors between test sessions. The number of entries to the open arm and the time spent in the open arm were recorded. Data were analyzed with Noldus software.

### Detection of cytokines

Rats were sacrificed after behavioral testing and blood plasma and brain were obtained. Parts of brain tissue were homogenized in a proportion of 1:10 (w: v) ice-cold saline and then centrifuged at 3000 rpm/min for 15 min at 4°C to get the supernatant. Protein concentrations were measured by using BCA (Pierce). Determination of cytokine concentration of interleukin-IL-1β (IL-1β*)*, IL-2, interferon-γ (IFN-γ) and tumor necrosis factor (TNF-*a*) was performed immediately according to the manufacturer’s instructions using a standard ELISA test and detected by spectrometer (TU1901, Purkinje General Limitid Coporation, Beijing) at 450 nm.

### Immunohistochemistry

Immunohistochemistry with Doublecortin (DCX) was conducted as previously reported [12]. Briefly, after behavioral testing, half of the brains were fixed by 4% paraformaldehyde in PBS, pH7.4 at 4°C for 48 hours. They were transferred to 30% sucrose and then cut with a Microm freezing microtome at a thickness of 20 μm and stained with progenitor neuronal markers DCX (anti-rabbit, 1:100, Santa Cruz) overnight at 4°C. After washing with PBS, the sections were incubated with Alexa 488-conjugated anti-rabbit IgG (1:500, Cell Signaling) followed by counter-staining with DAPI and then cover-slipped with anti-fade mount solution (Molecular Probes). Images were acquired with a Zeiss LSM PASCAL confocal microscope.

### Statistics

Results were presented as mean ± SEM. Differences among experimental groups were analyzed by one-way ANOVA with SNK-Q test for post-hoc multiple comparisons (version 16.0, SPSS Inc., Chicago, IL). P <0.05 was considered significant.

## Results

### Upregulated activity of mTOR signaling pathway in the developing brain in rats

**Fig 1:**
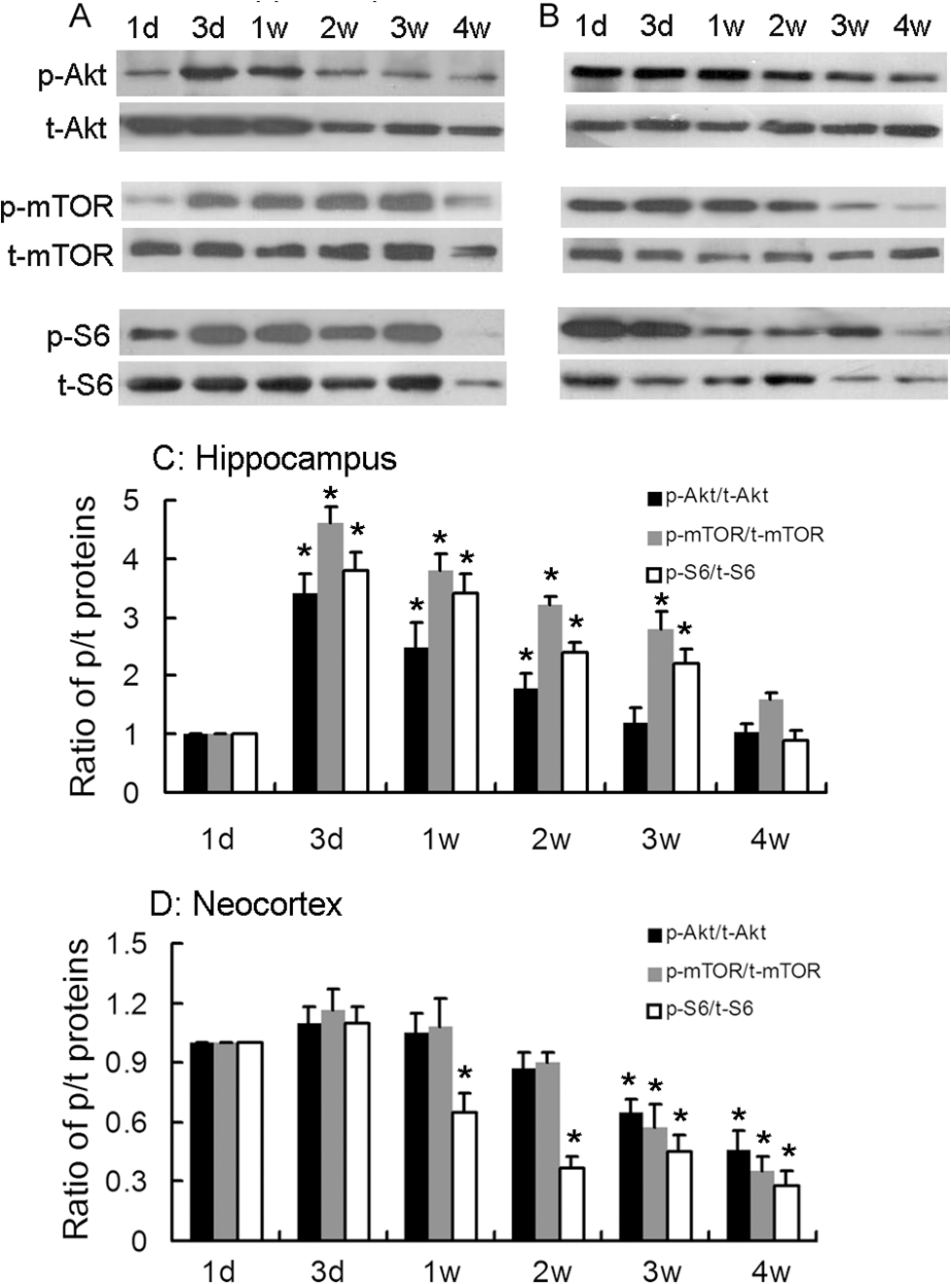
The key proteins of mTOR pathway are developmentally regulated. Representative blots of Western blot analysis of Akt, mTOR, S6 and their phosophorylation forms during brain development in hippocampus (A) and in neocortex (B). C. Quantitative summary of individual proteins in hippocampus demonstrated that p-Akt, p-mTOR and p-S6 were low at postnatal 1 d and transiently increased from 3 d to 2 w (p-Akt) or to 3 w (p-mTOR, p-S6). D. Quantitative summary of individual proteins in neocortex demonstrated that p-Akt, p-mTOR and p-S6 remained at a high level for the first 2 postnatal (p-S6) or 3 weeks (p-Akt and p-mTOR) and decreased thereafter. * p<0.05 compared to postnatal 1 d. n = 6 rats/ group.

We first analyzed the change of three key target proteins Akt, mTOR, and S6 in rats at different ages of 1 d, 3 d, 1 w, 2 w, 3 w and 4 w. The protein expression in hippocampus (Fig 1A and 1B) was slightly different than neocortex (Fig 1C and 1D) during brain development. In hippocampus, phospho-Akt was low at 1 d, increased to higher levels from 3 d to 1 w, and then decreased again at 2 w. The expression patterns of p-mTOR and p-S6 were similar, which were elevated from 3 d to 3 w, and then decreased when approaching adult age at 4 w. In neocortex, the expression of p-Akt, p-mTOR and p-S6 were the highest within postnatal 2 w and then slowly decreased thereafter. These results demonstrate that the activity of mTOR signaling pathway activity is transiently elevated during early brain development which is coincident with synaptogenesis.

### Short term treatment with rapamycin inhibited S6 phosphorylation in a dose-dependent fashion in young rats

**Fig 2:**
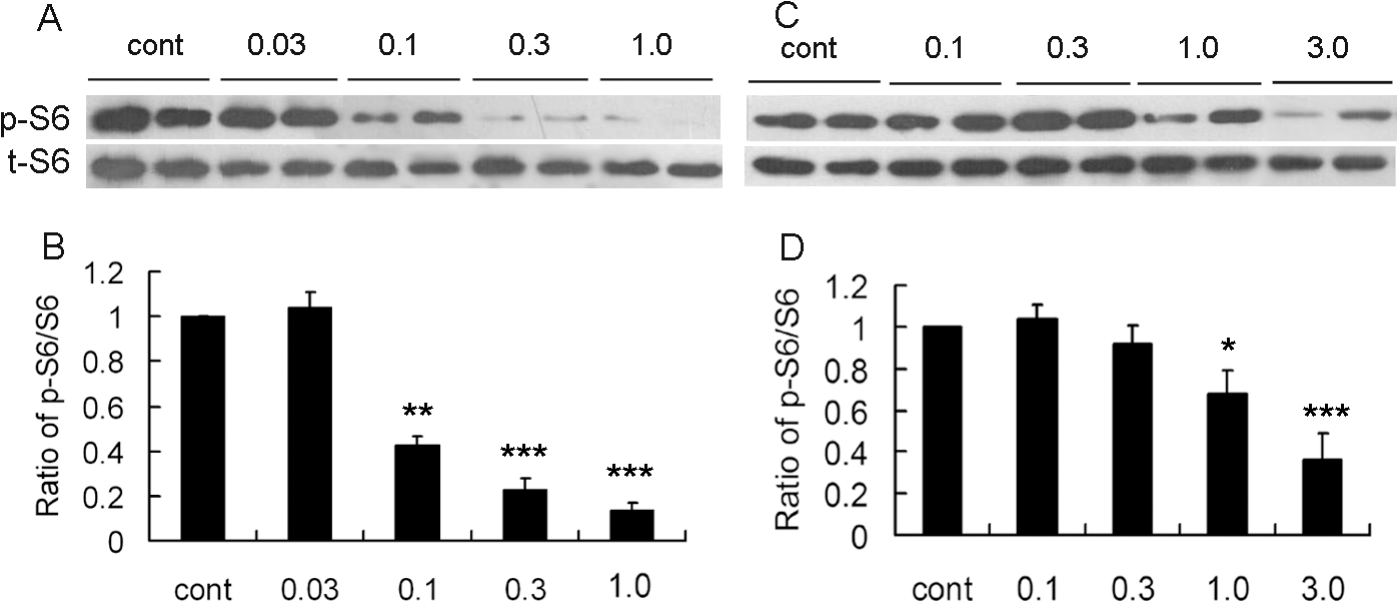
Rapamycin inhibits S6 phosphorylation dose-dependently in young rats. Rats of 2 weeks of age were administrated rapamycin for 3 d (A, C) or for 4 w (B, D). Representative blots in neocortex of short- (A) or long- treatment (B) with rapamycin at different doses. C. Quantitative summary demonstrated that 3 days treatment of 0.03 mg/kg rapamycin had no effect on p-S6, whereas 0.1, 0.3 and 3.0 inhibited p-s6 dose-dependently. D. Quantitative summary of 4 weeks treatment demonstrated that only 1.0 and 3.0 mg/kg rapamycin inhibited p-S6. *p<0.05, **p<0.01, ***p<0.001 compared to control group. n = 6 rats/ group.

We then treated rats of 2 weeks old of age with different doses of rapamycin for 3 days and analyzed its effect on mTOR signaling pathway as assayed by S6 phosphorylation in hippocampus. As we have observed a robust inhibitory effect at the dose of 3.0 mg/kg in 2 week old mice [7], here we tried relatively low doses of rapamycin at 0.03, 0.1, 0.3, and 1.0 mg/kg. While 0.03 mg/kg rapamycin had no effect on p-S6, rapamycin with the dose of 0.1, 0.3 and 1.0 mg/kg exhibited a dose-dependent inhibition of p-S6 in young rats and a marked inhibition was noticed at 0.3 (p<0.001) and 1.0 mg/kg (p<0.001) (Fig 2A and 2B). This result was different from our previous study in adult rats, which displayed significant inhibitory effects of rapamycin only at doses greater than 1.0 mg/kg [13].

### Effect of long term treatment of rapamycin on S6 phosphorylation in young rats

Next, we treated rats of 2 weeks of age for a relatively long time period of 4 weeks and assessed its effect on p-S6. In contrast to short-term treatment, we found low dose of rapamycin for 4 weeks had limited effects on S6-phosphorylation. The decrease of p-S6 was only noticed in rats treated with 1.0 and 3.0 mg/kg rapamycin (p<0.05) (Fig 2C and 2D).

### Effect of long term treatment of rapamycin on spleen, thymus and body weight in young rats

**Fig 3:**
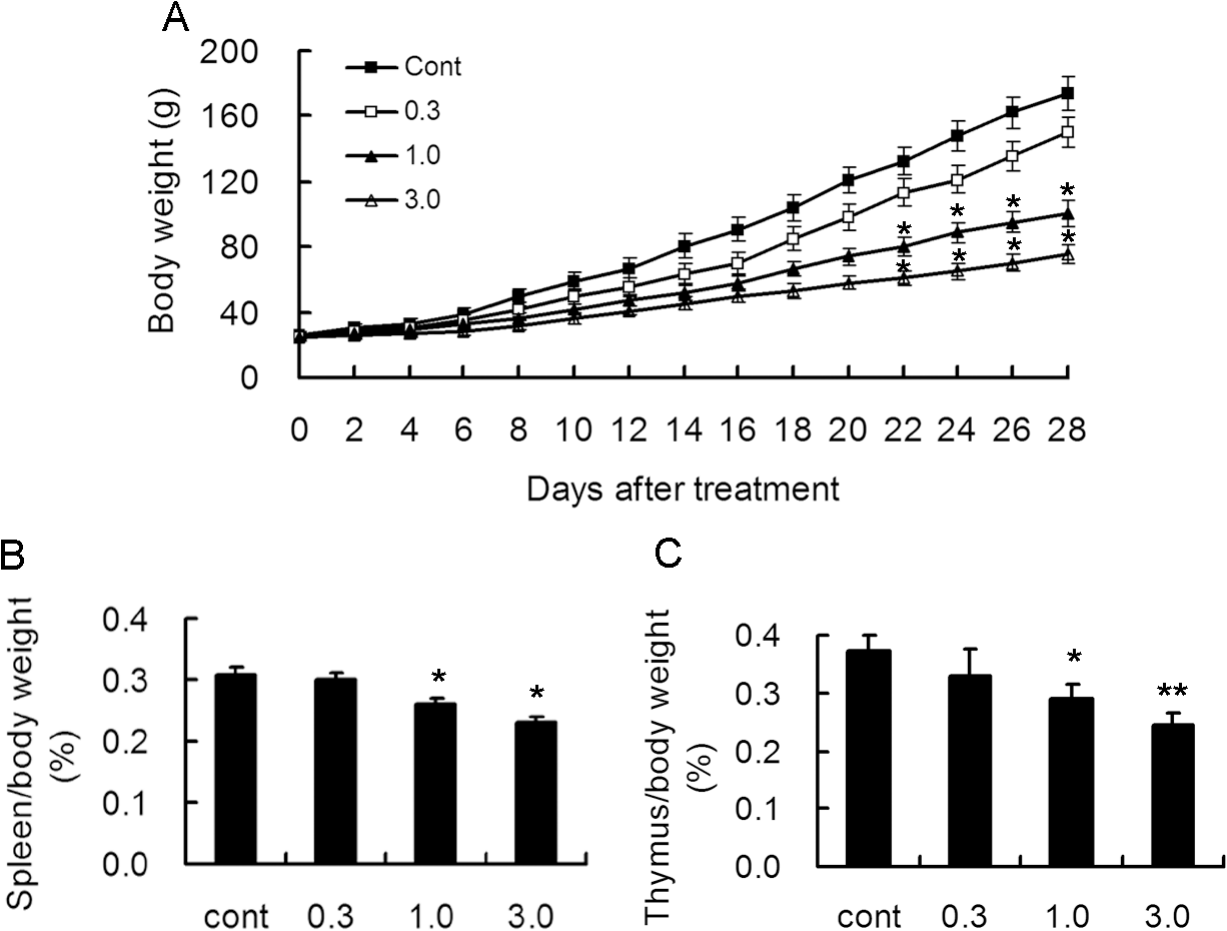
Long-term treatment with rapamycin inhibited the growth of body weight and immune organs in young rats. Rats of 2 weeks of age were administrated different doses of rapamycin for 4 weeks. A. Body weight monitoring demonstrated that treatment with rapamycin at 1.0 and 3.0 mg/kg decreased body weight significantly as compared to control rats 3 weeks after treatment. B. Rats were sacrificed after 4 weeks of treatment and the ratio of spleen to body weight was calculated. Rats treated with 1.0 and 3.0 mg/kg exhibited the ratio decrease. C. Rats were sacrificed after 4 weeks of treatment and the ratio of thymus to body weight was calculated. A decreased ratio was also found in rats treated with 1.0 and 3.0 mg/kg rapamycin. *p<0.05; **p<0.01 compared to control group. n = 15 rats/ group.

To confirm the effect of long term treatment of rapamycin during development, we monitored body weight during the 4 weeks of rapamycin treatment. Compared with the control group, rapamycin-treated groups at the dose of 0.3 mg/kg had slightly decreased weight gain, However, no significant difference was noticed. On the contrary, rats treated with 1.0 and 3.0 mg/kg rapamyin had an obviously delayed weight gain after 3 weeks of treatment. (Fig 3A).

As rapamycin is widely used in patients as an immunosuppressant drug, it is reasonable to consider whether long term administration of rapamycin had any effect on immune organs. Thus, we measured the weight of spleen and thymus after 4 weeks treatment and calculated the ratio of spleen and thymus to body weight. Compared with the control group, no inhibitory effect was found in 0.3 mg/kg rapamycin treated groups. However, both ratios were significantly decreased in 1.0 and 3.0 mg/kg rapamycin treated groups, suggesting potential inhibition of rapamycin on immune system in young rats even at relatively low dosage (Fig 3B and 3C).

### Effect of long term treatment of rapamycin on cognitive functions in young rats

**Fig 4:**
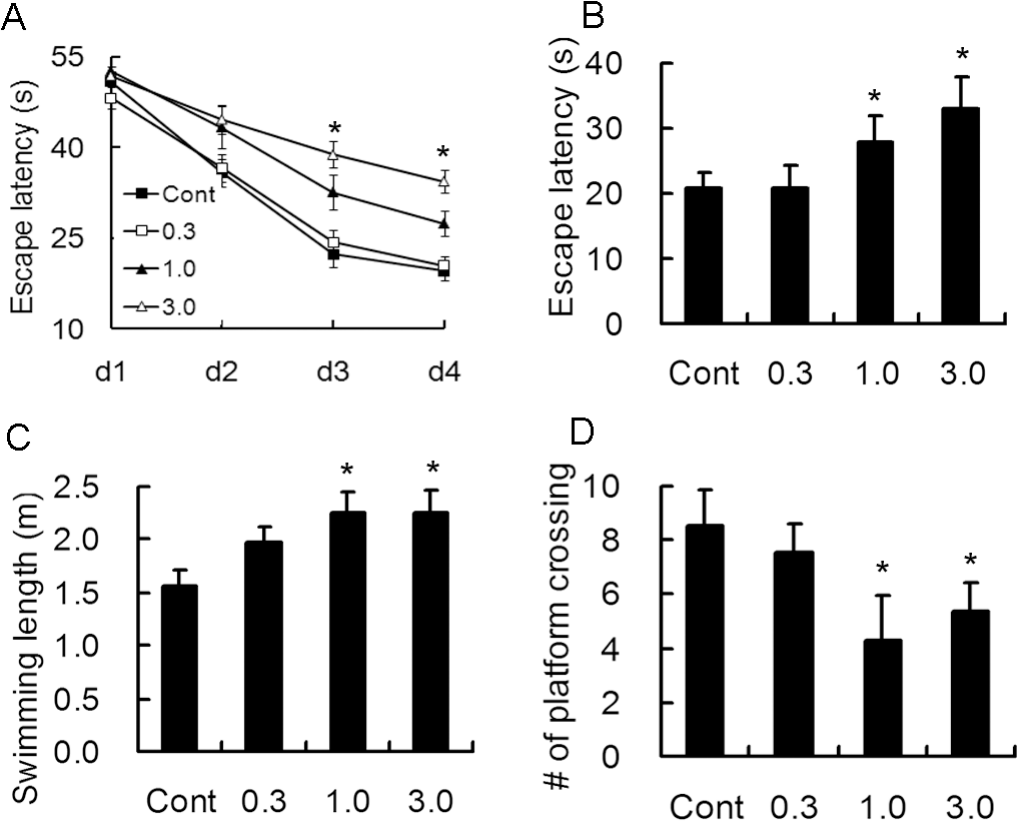
Long-term treatment with rapamycin affects spatial learning. Rats of 2 weeks of age were administrated different doses of rapamycin for 4 weeks and subjected to Morris water maze experiment. A. Rats injected with 1.0 and 3.0 mg/kg rapamycin had prolonged escape latency during training days 3 and 4. Rapamycin-treated rats at the dose of 1.0 and 3.0 mg/kg resulted in significant increase in escape latency (B) and swimming length (C) and decrease in number of crossing the target (D) on the 5th day. *p<0.05 compared to control group. n = 15 rats/ group.

Since mTOR signaling pathway is involved in synaptogenesis, another possible side effect of rapamycin may be its impact on cognitive function. Morris water maze and Y maze in rats were conducted after 4 weeks treatment with rapamycin. Consistent with the previous results, rats treated with 0.3 mg/kg rapamycin did not exhibited obvious cognitive impairment. However, rats treated with 1.0 and 3.0 mg/kg had obvious prolonged escape latency and swimming length in Morris water maze (Fig 4).

**Fig 5:**
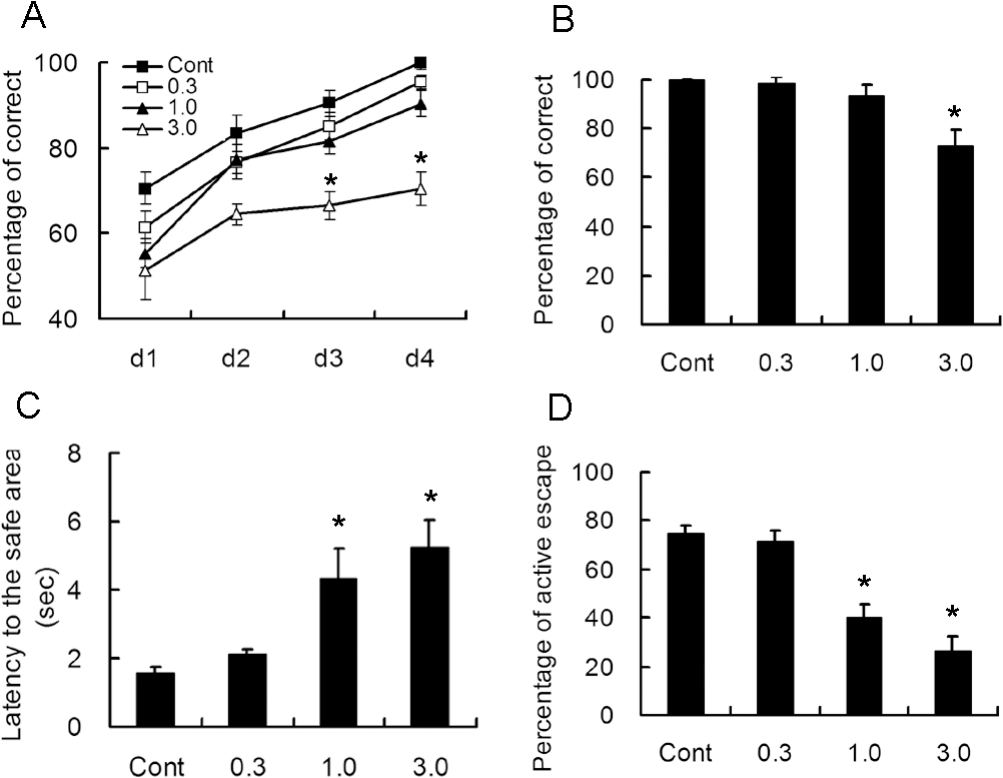
Long-term treatment with rapamycin results in cognitive impairment. Rats of 2 weeks of age were administrated different doses of rapamycin for 4 weeks and subjected to Y maze experiment after Morris water maze. A. The percentage of correct times during the training. It was only decreased in rats treated with 3.0 mg/kg rapamycin on the 3rd and 4th day. B. The percentage of correct times on the 5th day. C. The average latency to the safe area was markedly increased in 1.0 and 3.0 mg/kg rapamycin-treated rats. D. The percentage of active escape before electric shock was significantly decreased in 1.0 and 3.0 mg/kg rapamycin-treated rats. *p<0.05 compared to control group. n = 15 rats/ group.

Similar results were also found with Y maze testing. A significant difference in the percentage of correct was only noticed in 3.0 mg/kg rapamycin-treated rats during the training and 5^th^ day, however, both 1.0 and 3.0 mg/kg rapamycin-treated rats exhibited increased latency to the safe area and decreased percentage of active escape, which the rats ran before the electric shock (Fig 5).

### Long term treatment of rapamycin resulted in anxiety in young rats

**Fig 6:**
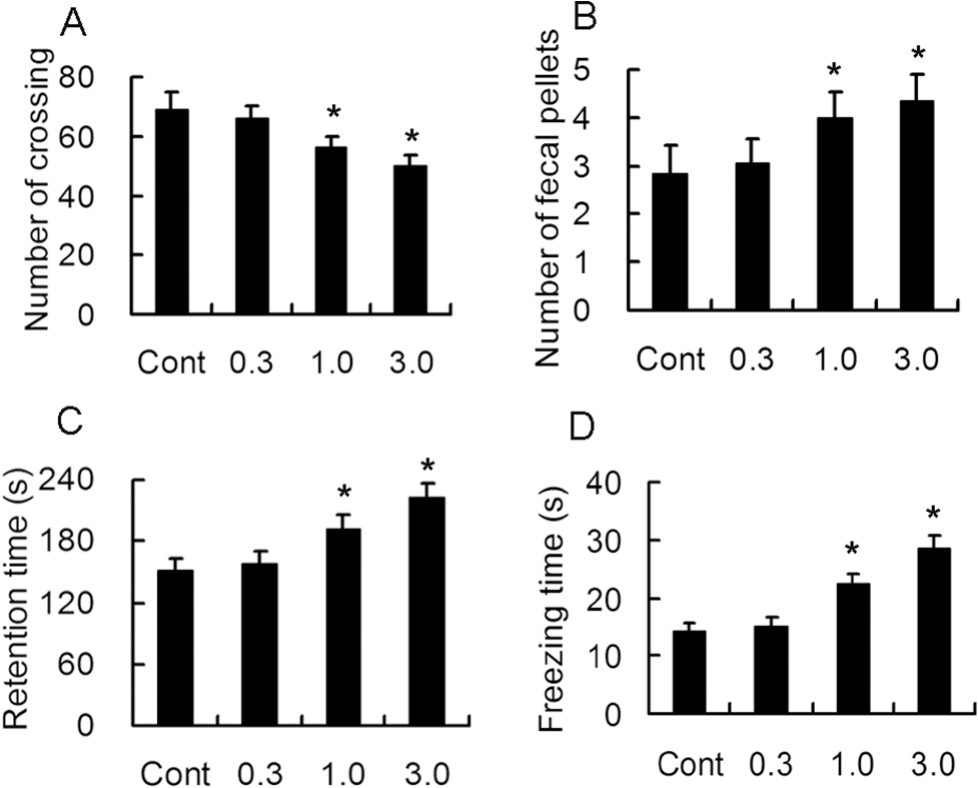
Long-term treatment with rapamycin results in anxiety-like behavior. Rats of 2 weeks of age were administrated different doses of rapamycin for 4 weeks and subjected to open field experiment after Y maze. Rats treated with 1.0 and 3.0 mg/kg rapamycin demonstrated decreased number of crossings (A), increased fecal pellets (B), retention time in the central area (C) and freezing time (D). *p<0.05 compared to control group. n = 15 rats/ group.

In open field experiments, the number of crossing, rearing, fecal pellets and retention time in the central area were analyzed and summarized in Fig 6. No change of number of rearing was noticed in all groups. Rats treated with 1.0 and 3.0 mg/kg rapamycin exhibited decreased number of crossings, increased number fecal pellets and retention time. These results suggested that long term treatment of rapamycin resulted in increased anxious behavior in young rats.

### Effect of long term treatment of rapamycin on cellular immunity in young rats

**Fig 7:**
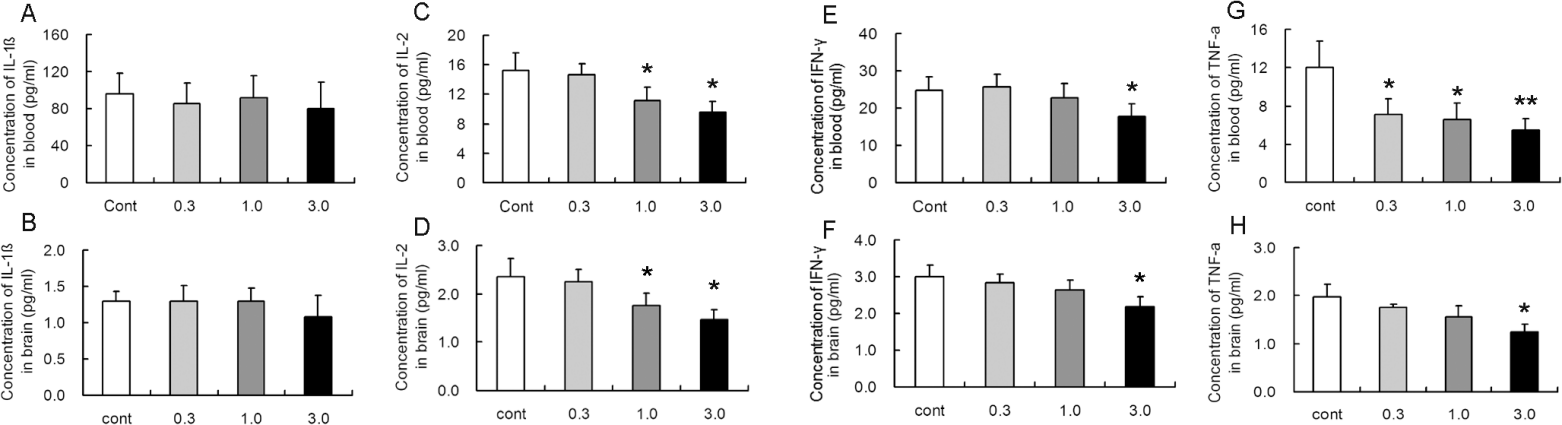
Long-term treatment with rapamycin results in decrease in cellular immune factors. Rats of 2 weeks of age were administrated different doses of rapamycin for 4 weeks and sacrificed. Cortical brain tissue (A, C, E, G) and blood (B, D, F, H) were collected and used for ELISA examination of Il-1β, IL-2, IFN-γ and TNF-α. No obvious change was noted among different groups in Il-1β both in brain and blood (A,B). IL-2 was decreased with 1.0 and 3.0 mg/kg rapamycin treatment both in brain and blood (C, D). IFN-γ was only decreased with 3.0 mg/kg rapamycin treatment both in brain and blood (E, F). All rapamycin-treated groups exhibited a decrease in TNF-α in brain (G), while the decrease was only shown in 3.0 mg/kg of rapamycin treatment in blood (H). *p<0.05; **p<0.01 compared to control group. n = 6 rats/ group.

Concerning the potentially inhibitory effect of rapamycin on cellular immunity, we examined the content of IL-1β, IL-2, TNF-α and IFN-γ both in blood and brain tissue. No differences of IL-1β concentration were noticed among different groups in blood and brain (Fig 7A and 7B). However, a marked decrease of IL-2 concentration was observed both in blood and brain in 1.0 and 3.0 mg/kg rapamycin treated rats (Fig 7C and 7D). Decrease of IFN-γ was only observed in 3.0 mg/kg rapamycin treated rats both in blood and brain. (Fig 7E and 7F). However, treatment of rapamycin induced a dose-dependent decrease in TNF-α in blood (Fig 7G), while decrease of TNF-α level was only detected in brain at the dose of 3.0 mg/kg (Fig 7H). These results were consistent to some extent with the effects on spleen and thymus.

### Effect of long term treatment of rapamycin on dentate development in young rats

**Fig 8:**
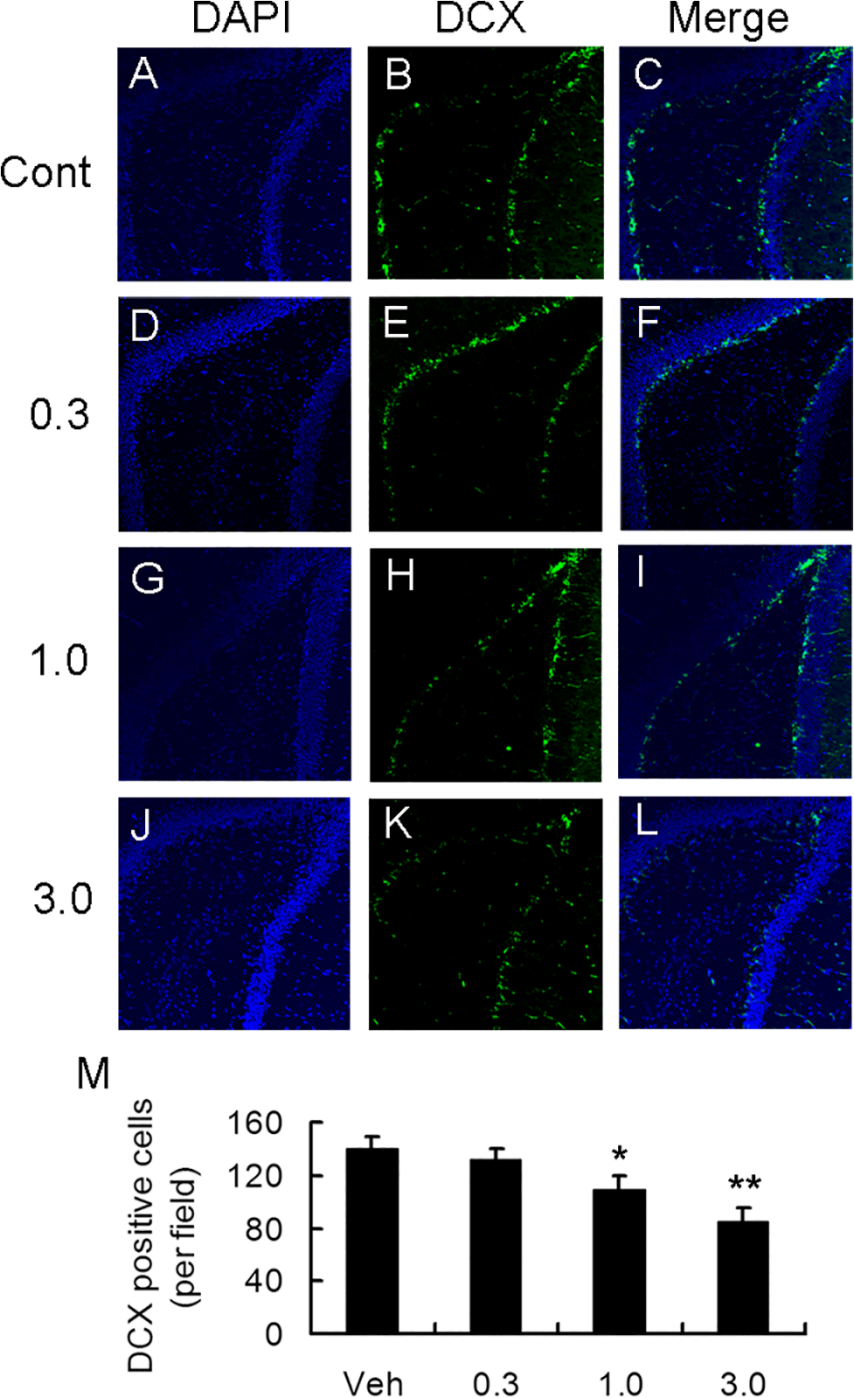
Long-term treatment with rapamycin results in decrease in progenitor cells. A-L. Representative images of immunohistochemical staining of DAPI, DCX and their merge. M. Quantitative calculation of DCX-positive cells per field in different groups. Rats treated with 1.0 and 3.0 mg/kg rapamycin demonstrated decrease in DCX-positive cells. *p<0.05; **p<0.01 compared to control group. Scale bar = 100 um. n = 6 rats/ group.

We also assessed the effect of rapamycin treatment on the development of late progenitors (DCX-expressing cells) after 4 weeks of rapamycin treatment. Similar to other findings in the present study, rapamycin at the dose of 0.3 mg /kg had no obvious effect on the DCX-positive cells, whereas 1.0 and 3.0 mg/kg rapamycin markedly decreased the DCX-positive cells in dentate gyrus in hippocampus (Fig 8). Therefore treatment with rapamycin at an early age decreased the development of late progenitor cells, which may exert adverse effects on development and subsequently affect cognition.

## Discussion

Rapamycin (or Sirolimus), originally isolated from a soil sample in Rapa Nui, is a macrocyclic fermentation product of Streptomyces hygroscopicus. It was initially investigated as the drug for the treatment of fungus and tumor. However, after its lymphopenic properties were discovered since 1997, it was widely used as an immunosuppressant in skin, heart, kidney and pancreas transplantation. Recently, the potential side effects of rapamycin and its analog everolimus have been thoroughly studied both in animal models and in patients suffering from transplantation and cancer. For example, dogs that received rapamycin at a relatively low dose of 0.05 mg/kg/day develop anorexia, fever, vomiting, leukocytosis and hyperamylasemia. At higher doses (0.3 to 2.0 mg/kg/day), submucosal vasculitis with mucosal erosion/ulceration has been observed [16]. Additionally, myocardial necrosis has been reported in rat models [17]. Human patients administrated rapamycin at low daily doses (1 to 2 mg/day) develop various side effects including headaches, polyarthralgia, mild stomatitis, epistaxis, diarrhea, and skin acne [18–20]. Of course, the major concerns of rapamycin administration are myelosuppression, hyperlipidemia, and immunosuppression—related problems.

Since the finding that the tuberous sclerosis genes *TSC1* and *TSC2* are suppressors of the mTOR signaling pathway, mTOR inhibitors have been used as a treatment approach in tuberous sclerosis. The efficacy and safety of rapmycin and everolimus have been investigated in young patients as well as animals. In a multicentre, randomized, placebo-controlled phase 3 trials for everolimus in patients aged 0–65 years using the Common Terminology Crieria for Adverse Events, adverse effects were reported mild and well-tolerated. The most frequent adverse events were infections as pneumonia and stomatitis [21]. Another report using everolimus as a treatment for subependymal giant cell astrocytoma associated with tuberous sclerosis complex for approximately 3 years found the most frequent adverse events were also aphthous stomatitis and respiratory infections in children under 3 years of age, and no significant neuropsychological changes were noticed between baseline and follow-up [22]. In animal models, although chronic rapamycin treatment from postnatal day 8 to 40 improved anxiety- and depression- like behaviors in a model of Tsc1 [23], daily rapamycin administration beginning at E12.5 resulted in hippocampal-dependent learning impairment in a mice model of Tsc2 [24]. In addition, one dose administration of rapamycin at embryonic day 16.5 in wildtype of C57Bl/6 mice resulted in sensorimotor impairments and increased anxiety-like behaviors [25]. Thus, the observed discrepancy in adverse effects may be due to the different animal model and timing of drug treatment. Our study adds significance related to the potentially detrimental effects of rapamycin in young animals.

To identify a suitable dose which can block the overactivation of mTOR but not interrupt the baseline activity in young rats, we first detect the expression of the markers of mTOR signaling during brain development. We found that phosphorylation of Akt, mTOR, and S6 are transiently increased in postnatal 3 weeks. This period is the time for brain development as well as synaptic plasticity. In hippocampus, the expression of phosphorylated Akt was highest during postnatal 3 d to 1 w. Interestingly, there was a developmental time lag between the increase in phospho-Akt and its downstream target phospho-mTOR and phospho-S6. In neocortex, the expression of phosphorylated Akt was highest within postnatal 2 weeks and decreased thereafter. To date, this is the most detailed description of the mTOR signaling pathways during development in SD rats, and is a little different from what was reported in Long-Evans rats [11]. Many reports have shown that the activity of mTOR is highly regulated and balanced. As previously reported, S6 phosphorylation is regulated by other upstream signaling pathway besides mTOR itself, such as PI3K-Akt, and is subjected to multiple positive and negative feedback loops. This negative feedback loop might be especially strong during development.

Combined with our previous data and the above findings, we set the dose of rapamycin from 0.03 to 3.0 mg/kg and determined their effect on mTOR pathway both acutely and chronically. Results of short-term treatment revealed that 0.1, 0.3 and 1.0 mg /kg rapamycin exhibited a dose-dependent inhibitory effect, however, only 1.0 mg/kg rapamycin inhibited phospho-S6 after four week treatment. One explanation is that low dose rapamycin has inhibitory effect only when the mTOR signaling pathway is highly activated before postnatal 3 weeks. More time points should be examined to determine how long low dose rapamycin will maintain its inhibitory effect. Another study is now being conducted to test whether low dose rapamycin is effective against epilepsy in a mouse model of infantile seizures.

The link between mTOR and synaptic formation has been investigated extensively [26,27]. Synaptic plasticity is the alteration of the strength of connections which changes with the synaptic activity. Long-term potentiation and long-term depression of synaptic transmission, the two main formations of long-term synaptic activity, are widely recognized as the molecular mechanism of learning and memory. Protein synthesis is important for synaptic plasticity. Although a few reports have shown that rapamycin prevents status epilepticus-induced cognitive deficits in adult rodents [28–30], and even extend life–span in elderly mice [31, 32], long-term administration of relatively high dose of rapamycin impairs learning and memory [33]. In the present study, we found that 1.0 and 3.0 mg/kg rapamycin administration impaired cognitive functions, as well as caused increased anxiety. Future behavioral studies may help differentiate effects on memory versus emotional function. We also found the progenitor cells were decreased in 1.0 and 3.0 mg/kg rapamycin–treated rats as evident by immunohistochemical staining of DCX, which are important in the developing brain as they increase the number of functional neurons which is important to maintain cognition and memory. Postnatal neurogenesis continues throughout life in DG of the hippocampus, and this ongoing neurogenesis relates closely to the mTOR pathway. Additionally, reports have shown that long-term treatment with rapamycin is associated with a decrease in the number of astrocytes and microglia [34]. Future studies are needed to investigate the expression of other cells during brain development in young rats.

In the adaptive immune system, mTOR can be activated by the stimulation of T cell receptor and cytokine receptors. In addition, mTOR regulates cell cycle progression from G1 into S phase in cytokine-stimulated T cells and natural killer cells. Hence, it is reasonable that rapamycin potently decreases the production of cytokines. In the present study, we found that the weight of thymus and spleen was relatively decreased in rapamycin-treated rats. Furthermore, rapamycin at 1.0 and 3.0 mg/kg administration decreased the concentration of IL-2 and TNF-alpha both in blood and brain. These results are consistent with those reported [35,36,37], and the inhibition effect of rapamycin on immune system may be responsible for the adverse effect of opportunistic infections, such as pneumonia which is the most common adverse effect in transplant patients.

The adverse effects of long- term treatment of rapamycin have been explored other than development in young animals. For example, adult male SD rats treated with rapamycin for 15 days exhibits increased glucose intolerance and insulin resistance [38]. Future experiments should address the effect of rapamycin in young rats, relative to the other effects observed in the present study. Paradoxically, rapamycin may extend lifespan or preserve memory in aging animals. Since mTOR signaling pathway has multiple downstream targets and is also regulated by upstream signaling, inhibiting mTOR pathway by rapamycin may exert different effect by targeting various downstream signaling. In aging animals, rapamycin may extend lifespan or preserve memory by controlling metabolism, activating NO synthase or enhancing NMDA signaling [39–41].

In summary, the present data show that rapamycin at the dose of higher than 0.1 mg/kg suppresses mTOR signaling pathway for short periods and higher than 1.0 mg /kg suppresses mTOR signaling pathway chronically in young rats. Long treatment for 4 weeks with rapamycin higher than 1.0 mg/kg results in cognitive and immune function impairment, indicating that the lowest dose that is effective in mTOR signaling inhibition over 4 weeks also has side effects. However, 1 mg/kg is the optimal dose since it maintains efficacy while minimizing side effects. Caution should be used when long-term rapamycin is administered to young patients, and adverse effects associated with cognitive impairment and immunosuppression should be monitored. More detailed studies, however, are required to investigate the effects of rapamycin therapy at additional developmental time points and also in animal models of epilepsy on neurocognitive development.
